# Examining the Complexity of Patient-Outpatient Care Team Secure Message Communication: Qualitative Analysis

**DOI:** 10.2196/jmir.9269

**Published:** 2018-07-11

**Authors:** Holly Jordan Lanham, Luci K Leykum, Jacqueline A Pugh

**Affiliations:** ^1^ Division of General and Hospital Medicine Department of Medicine University of Texas Health San Antonio San Antonio, TX United States; ^2^ Department of Information, Risk and Operations Management McCombs School of Business The University of Texas at Austin Austin, TX United States; ^3^ Veterans Evidence Based Research, Dissemination, and Implementation Center (VERDICT) South Texas Veterans Health Care System San Antonio, TX United States

**Keywords:** secure messaging, patient-physician communication, complexity science, outpatient care, outpatients, confidentiality

## Abstract

**Background:**

The value of secure messaging in streamlining routine patient care activities is generally agreed upon. However, the differences in how patients use secure messaging, including for communicating both routine and nonroutine issues, and the implications of these differences in use are less well understood.

**Objective:**

The purpose of this study was to examine secure messaging use to extend current knowledge of how this tool is being used in outpatient care settings and generate new research questions to improve our understanding of the role of secure messaging in the patient-provider communication toolbox.

**Methods:**

We conducted an in-depth qualitative analysis of secure message threads in 12 US Department of Veterans Affairs outpatient clinics in south Texas. We analyzed 70 secure message threads with a total of 179 unique communications between patients and their outpatient teams for patterns in communication and secure message content. We used theories from information systems and complexity science in organizations to explain our observations.

**Results:**

Analysis identified content relating to 3 main themes: (1) information management, (2) uncertainty management, and (3) patient safety and engagement risks and opportunities. Within these themes, we identified 2 subcategories of information management (information exchange and problem solving), 2 subcategories of uncertainty management (relationship building and sensemaking), and 3 subcategories of patient safety and engagement risks and opportunities (unresolved issues, tone mismatch, and urgent medical issues). Secure messages were most often used to communicate routine issues (eg, information exchange and problem solving). However, the presence of subcategories pertaining to nonroutine issues (eg, relationship building, sensemaking, tone mismatch, urgent issues, and unresolved issues) requires attention, particularly for improving opportunities in outpatient care settings using secure messaging.

**Conclusions:**

Patients use secure messaging for both routine and nonroutine purposes. Our analysis sheds light on potentially new patient safety concerns, particularly when using secure messaging to address some of the more complex issues patients are communicating with providers. Secure messaging is an asynchronous communication information system operated by patients and providers who are often characterized as having significant differences in knowledge, experience and expectations. As such, justification for its use beyond routine purposes is limited—yet this occurs, presenting a multifaceted dilemma for health care organizations. Secure messaging use in outpatient care settings may be more nuanced, and thus more challenging to understand and manage than previously recognized. New information system designs that acknowledge the use of secure messaging for nonroutine and complex health topics are needed.

## Introduction

### Background

Secure messaging is rapidly becoming a commonly used health information technology (IT) tool [1]. This electronic communication feature, embedded within a patient portal, allows patients to communicate privately and securely with members of their care team about their health and medical condition(s), as well as about administrative matters such as medication refills and appointment requests. Despite reports of provider apprehension that use of secure messaging would increase workload [2-6], both patients and providers increasingly regard this health IT tool as an effective way to streamline health care delivery [7-10]. Patients who use secure messaging report higher patient satisfaction, improved face-to-face visits, and improved access to care outside of traditional in-person clinical visits [11]. Providers report positive impacts of secure messaging as well, particularly in terms of streamlining medication refills, managing referral requests, and scheduling appointments [4,12].

### Features of Secure Messaging

Recent research has begun to highlight some important strengths of secure messaging tools for disease management, including a study showing that the use of secure messaging for prescription refills was associated with greater control of HIV viral load [13]. At the same time, studies identified key factors contributing to patient-level differences in secure message use, including end-user goals, internet availability, health literacy, and computer literacy [14]. At an organizational level, human resources, technology resources, and leadership support are associated with increased secure message adoption rates; higher secure message use is associated with lower urgent care use; and early adopters of secure messaging experienced a greater decrease in urgent care use over time than did later adopters [15]. Another study pointed to the perplexing nature of the IT-supported patient-provider relationship, finding that patients were responsive to provider engagement with secure messaging. Patients were more likely to use secure messaging if their provider frequently initiated messages to patients in general. If a provider was a low initiator, their patients were likely to be infrequent users of secure messaging as well [16].

Evidence of the value of secure messaging in streamlining routine patient care activities is growing [17-19]. While a substantial portion of the secure messaging literature has focused on describing the types of activities for its use, it has placed little emphasis on examining the complexity of these various activities and how different levels of message complexity might affect communication between patients and their providers. Secure messaging, because it is an asynchronous and virtual communication channel, is a lean form of communication lacking the capacity to convey the typical cues that characterize interpersonal conversation [20,21]. Gestures and nonverbal nuances, cues of social influence, symbolic content, and contextual cues are not easily captured and transmittable in secure messages. Thus, effective communication in an asynchronous technology-mediated context requires a great deal of effort and attention [22,23].

The effective transfer of rich information and the communication of ambiguous information via secure messaging is not well understood. Likewise, the linkage between secure message use and patient safety, and between secure message use and patient engagement, is not well understood. While researchers have examined secure message use in terms of message volume, frequency, and response time [12] and described activities for which secure messaging is used (eg, medication refills, appointment scheduling, referral request, and questions about medical conditions) [3,24,25], less is known about the nature of the information being exchanged and sought, and about capabilities of secure messaging for conveying information that is complex or nonroutine. We focused our examination on this aspect of secure messaging in an effort to extend current knowledge of how this tool can support outpatient care delivery, particularly for understanding the potential patient safety and engagement implications of using secure messaging to address nonroutine tasks and complex issues.

### Conceptual Framework

We used complexity science [26-29] to frame our analysis because of its emphases on examining the interdependencies between system elements [30-32] and uncertainty management [31,33]. Complexity science is a useful lens through which to study relationships among primary care providers [34,35], care improvement in nursing homes [36], and collaboration in intensive care units [37]. In addition to its application in studying a diversity of health care delivery settings, complexity science has been useful in examining provider-level differences in health IT use [31], examining clinic-level differences in the implementation of health IT for practice redesign [38], and developing a sociotechnical model for studying health IT in health care systems [32]. Complexity science helped us evaluate secure messaging interactions between patients and their outpatient care team as a system of relationships [39] sometimes characterized by high complexity (contexts that cannot be fully understood simply by analyzing individual components of the system) [27] and uncertainty (an inevitable and natural part of complex systems that cannot be avoided, eliminated, or controlled) [40,41]. Thus, our study was both theoretically driven and grounded in the reality of everyday technology-supported communication between patients and their care teams.

## Methods

### Study Design

We conducted a retrospective in-depth qualitative analysis of secure message threads sent between patients and provider teams in 12 US Department of Veterans Affairs (VA) outpatient clinics of a single VA health system in south Texas, USA. Study clinics consisted of 9 primary care clinics, 1 mental health clinic, 1 allergy clinic, and 1 geriatric evaluation and management clinic. The VA is undergoing a series of transformational initiatives to design a veteran-centric health care model and infrastructure to help veterans navigate the health care delivery system and receive coordinated care [42]. Health IT is a major part of this transformation, including the rollout of secure messaging as an additional communication channel for patients and their providers. This particular VA system uses a triage team model in its implementation of secure messaging: patients contact their care team and a nurse triages the messages, escalating messages to physicians as needed. We used a combination of thematic content analysis [43] and medical record audits. To follow up on any issues that appeared unresolved in the secure message thread, we examined patient medical records for signs of patient-provider communication that occurred through modalities other than secure messaging. The University of Texas Health Science Center San Antonio and South Texas Veterans Health Care System, San Antonio, TX, institutional review boards approved this study.

### Data Collection

From the 12 study clinics, we retrospectively collected all secure messages sent between May 19, 2013 and December 19, 2013. We selected this time period to allow us to collect and analyze an adequate number of messages. At the time of the study, secure messaging was in the early phases of rollout and implementation at this VA. The only inclusion criterion was that the message was initiated by a patient of 1 of the 12 clinics during this 7-month time period. Data collection, achieved via a query sent to a clinical systems analyst followed by manual review, resulted in 70 total message threads with 179 unique messages and between 5 and 8 message threads per clinic. Each secure message thread was initiated by a unique patient and contained between 1 and 7 unique messages between a patient and their care team. The messages were captured in Word, deidentified and printed out for analysis. We excluded no messages in the analysis.

### Analysis

We qualitatively analyzed [44] messages in 3 phases: (1) content analysis focused on uncovering general themes in the data and then developing subcategories under each theme, (2) systematic coding of the data, and (3) medical record auditing.

#### Content Analysis

The first phase was a content analysis using an open coding approach [45]. Two researchers (HJL, JAP) read and coded all messages. Messages were read to identify patterns in the types of information being exchanged or sought (requested). We abstracted text segments into a coding matrix to help with data sorting. During analysis, all 3 authors met to review and reach agreement on selected segments and the codes.

As themes emerged, we used complexity science literature in information systems and organizational sciences to interpret and refine themes and subcategories. For instance, medication renewals and appointment scheduling are message types that have been covered in the literature by previous studies of secure messaging, as information exchange and problem solving are known *information management* activities [46,47]. As such, we used this literature to define and examine these categories. We defined *information exchange* as content that is primarily aimed at sharing or transferring information between parties. We defined *problem solving* as content that presents a problem to be addressed.

We also identified patterns in the use of secure messaging that we coded as relationship building and sensemaking. For instance, we observed patients and providers who seemed to be using secure messaging as a way to establish or maintain the patient-provider relationship (relationship building). Further, we observed content in the messages where patients expressed confusion about their medical situation and sought help in interpreting or assigning meaning to something they were experiencing or to information they discovered from the patient portal or from another information source (sensemaking). Relationship building and sensemaking are known strategies for *uncertainty management* [33,40,48,49]. We used complexity science literature in organizational science and information systems [50,51] to define and examine these categories. We defined *relationship building* as content that sought to establish or maintain a relationship between parties. We defined *sensemaking* as content that demonstrated the seeking of new understanding or meaning, or help with interpretation of complex or ambiguous information.

Finally, we identified themes in the data pertaining to potential *patient safety* and *patient engagement* risks (or opportunities for improvement). For instance, we observed delays in care team responses to patient-initiated messages, tone mismatches between patient and care team messages, and urgent issues being communicated by patients via secure message. We viewed such content as having the potential to introduce unanticipated safety risks and detrimental effects on patient engagement. Therefore, we also coded the messages for these 3 safety and patient engagement subcategories. We defined *unresolved problems* as problems initiated in a secure message thread that were sometimes not resolved in that same thread and may have gone unresolved. We defined *tone mismatch* as messages from patients that included personal, emotional, or mental health details, or that were of a style that provided abundant detail; care team responses to these messages were brief or curt in tone, in contrast to the tone or content of patient-initiated messages. We defined *urgent medical issues* as messages that contained text with urgent or highly complex medical issues needing immediate medical attention.

#### Systematic Coding

In the second phase of analysis, we systematically coded the secure message threads for the 2 information management subcategories (information exchange, problem solving), 2 uncertainty management subcategories (relationship building, sensemaking), and 3 patient safety and patient engagement subcategories (unresolved issues, tone mismatch, urgent medical issues) that we had identified in the first phase of analysis [44]. Two authors (HJL, JAP) independently coded the messages and a third author (LKL) provided an additional perspective to resolve coding discrepancies and reach conceptual agreement. We discussed coding in 5 group sessions to ensure adequate consistency in the application of the coding definitions. Discussion with all 3 authors led to the final coding scheme, and all authors reviewed the final coding for consistency and accuracy. Because threads frequently contained multiple messages, some threads were coded for multiple categories. Longer and more complex unique messages were often coded for multiple categories.

#### Medical Record Auditing

In the third phase of analysis, 2 authors (JAP, HJL) conducted medical record audits to follow up on messages that appeared unresolved after analyzing the secure message threads and messages categorized as urgent. The goal was to determine whether the issue or issues raised via secure message was or were ultimately addressed outside of the original secure message thread (eg, via an office visit, scheduled subspecialty outpatient visit, phone call). To do this, we searched for and reviewed additional secure messages sent after the data collection period to see whether the issue in question was resolved in a subsequent message. We also searched the medical record for follow-up appointments and office visits related to the issue raised via secure message. For example, if a patient asked for a referral to a physical therapist and the issue was unresolved in the original thread, we looked for a physical therapy visit or appointment scheduled close to the original request made via a secure message. We examined the medical record for anything that would signal or provide data that the issue was ultimately resolved. In this step, we considered all subsequent secure messages, visits, consults, and phone calls within 3 months of the initial secure message to be potentially involved in resolving an issue that appeared to have been unresolved in the original secure message thread.

## Results

We identified and analyzed patient-outpatient care team secure message communication for 7 subcategories of secure messaging use nested within 3 main themes. We identified 2 information management subcategories, 2 uncertainty management subcategories, and 3 patient safety and engagement subcategories. Table 1 provides example quotes from the messages and the total number of threads coded for each category. Each quote was obtained from a unique patient. Approximately 50% (6/11) of the messages initially categorized as unresolved remained unresolved 3 months following the initial secure message communication.

### Information and Uncertainty Management Strategies

We categorized secure message content as information management (information exchange and problem solving) and uncertainty management (relationship building and sensemaking). Information exchange (37/70, 53%) and problem solving (29/70, 41%) were more prominent in the data than sensemaking (10/70, 14%) and relationship building (6/70 instances, 8.6%).

### Patient Safety and Patient Engagement Risks and Opportunities

We categorized secure message content related to patient safety and engagement as resolved or unresolved, matched or unmatched in tone between the patient’s secure message content and the outpatient care team’s secure message content, and urgent or nonurgent. We observed instances (11 out of 70) where issues raised over secure message appeared unresolved. It was not possible to tell from the original thread whether the problem was resolved in another thread, for example, or by a phone call or face-to-face visit—or if the problem truly went unresolved.

We observed tone mismatches between patients and outpatient care teams in 11 out of 70 messages. This most often occurred when a patient provided rich or personal details in their message to the care team and the care team responded using a template-type response, such as “Noted, will forward this to your provider.” Other times, care team responses to highly emotional messages from patients were brief and curt in tone.

A less frequent type of message (3 out of 70) contained urgent medical questions from patients to their outpatient care teams (eg, seriously out of control blood pressure, suicidal thoughts). This observation suggests the need for further examination of the circumstances under which patients decide to use secure messaging for urgent medical matters, a growing patient safety concern [52].

Medical record reviews found that approximately 50% (6/11) of the messages categorized as unresolved remained unresolved after the medical record review. Issues that were resolved were addressed by phone or in-person visit. Of the 3 urgent secure messages, we also categorized 1 as unresolved and it appeared to remain unresolved after the medical record audit. The other 2 urgent messages were resolved by phone.

The findings generated from the medical record review supplemented the findings from coding analysis. Additionally, this step provided further insight into potential patient safety risks involved in unanticipated secure messaging use by patients.

**Table 1:** Secure message use subcategories and exemplar quotes in 70 message threads.

Subcategory	n (%)	Example content
Information exchange	37 (53)	*...would like to request a consult to be placed with physical therapy for “dolphin stem” treatment to help with scar tissue buildup post total knee replacement. Thank you.* *I have tried to call and cancel throughout the weekend with no avail. I will not be able to make my appointment this afternoon. Please be sure to cancel it for me. Thank you.*
Problem solving	29 (41)	*I have a nasty head cold. My nose is running constantly, sneezing, ache all over. Is there any over the counter meds I can take to help that won’t react with the medications I’m taking?* *...my omeperzole [* sic*] has change there [* sic*] giving only filled my last script with 1 cap 10 mg a day. I need my coverage [3 times a day] due to my frequent feeding cause of my gastric bypass surgery. I have heart burn without why was it changed. Also can you send prosthetic a script for diabetic shoes they said my last script expired. Thank you.*
Relationship building	6 (8.6)	*I would like to set up this line of communication so that my appoints in the future will not be overlooked. Also I would like to apologize to _____ staff for my forgetting and missing my 11/19/13 appointment. Now that I have access to this Web page all my important information is in one place. Sincerely, _____* *Hey there young man. You all ready for Christmas? If you are, you got me beat. _____, you didn’t do a dam thing wrong my friend. Something was blocking the messages from coming through to me, that’s it. Now as you can tell, everything is back to working just fine. Thank you for your help and patience.*
Sensemaking	10 (14)	*Hi Team _____, I had a [computed tomographic] scan of my chest last week and was able to look at the results online.* Saw some words that make me uneasy, can you give me a quick email with your impressions and summary? *Dr _____, I just wanted you to know that I had my Methacholine challenge test yesterday.* I was confused when *the tech said it showed I DON’T have asthma. I was wheezing and a 72-year-old lifetime smoker by the last test. Then she gave me a dose of Albuterol, which cleared me right up and enabled me to blow the last spirometry test away. It that tightness and wheezing was not asthma, then what was it? I know we’ll be able to talk about this next week at our appointment.* *The reason I kept going to my mental health doctor, was not because I wanted to, it was because I needed to. I have serious problems with depression. I cry for no reason and have thoughts of suicide, I just want to lay in bed and do nothing, and I don’t even want my son (who I love with all my heart) around me. I am taking Fluoxetine on a regular basis now and I’m still having bouts of depression. I really wasn’t relaying this very well with my doctor, mainly because I wasn’t having a “dark day” when I saw him. I need something to help with all these bouts that I have. It’s an ongoing thing. Please help. I don’t know why I keep having these.*
Unresolved problems	11 (16)	*I have been seeing double vision 3 or 4 times every day for 2 to 4 minutes each time. For the past week I have been getting light headed just doing chores. My carpal tunnel supports need replacement please both of them. Thank you.* *Response: none*
Mismatches in tone	11 (16)	Long, detailed, multiproblem message with short response from patient-aligned care team nurse: “Will forward your concern to the doctor.”
Urgent medical issues	3 (4.3)	*Dr ____, This morning I was to have an endoscopy but it was cancelled due extremely high blood pressure. I am faithfully taking my meds each morning around 9-9:30. I took the pills as directed this morning at 6am and arrived at the VA around 6:45am. My blood pressure was 208/110 and came down to 186/100 and then back up to the 200+ range. The endoscopy was cancelled. The chief of endoscopy was quite concerned as I was because I took my meds and have been taking them like I said – every morning. Now, I have had a lot of stress in the last 3 weeks. My father died and my brother and I are trying to get things…* Patient then goes on to describe death in the family plus other somber matters.

## Discussion

### Principal Findings

In this qualitative analysis of secure message communication between patients and providers in 12 outpatient clinics, we observed patterns in message content relating to secure message type and purpose beyond previously reported barriers and facilitators of secure message use, impacts on clinical workflow, and impacts on efficiency. We found secure message content to be straightforward and unambiguous in most messages. Patients used secure messaging as one might expect: for example, to check the status of a laboratory test result or to request a medication renewal. However, many messages were complex and multipurposed, often containing nonmedical, personal, or contextual details about the patient’s life and social or personal situation. Others contained ambiguous or more complicated, less routine medical content that may not be easily addressable with a lean communication tool such as secure messaging [20].

Our analysis generated new questions about the use of secure messaging for nonroutine health care tasks and about how patients and care teams use secure messaging to communicate more complex and ambiguous information. Some patients shared highly personal and emotional content in their messages, others expressed discomfort with uncertainty in their medical condition, and a few patients conveyed urgent medical matters to their outpatient care teams via secure messaging despite being advised against it. Provider concerns about these issues are not new. Studies describe provider concerns about messages that may be long, vague, and difficult to answer or inappropriately urgent [53-58]. Our study, however, provided evidence that such provider concerns are valid. This finding is important, as the tendency may be to accuse providers of expressing “concern” as a way to avoid using secure messaging tools for communicating with patients. Are secure messaging platforms set up to manage the exchange of information relating to more complex, nonroutine issues? If not, what interventions can be made from an organizational- or policy-level perspective to improve the ability of secure messaging platforms to communicate this type of information or to help manage the risk to patients’ health if urgent matters are inappropriately communicated via secure messaging?

We also found tone mismatch and unresolved issues in our data, showing these lapses as examples of ineffective secure message patient-provider communication. Our findings generated new knowledge about the content of secure message conversations between patients and their providers and suggest potential links between secure message use and patient safety and patient engagement.

### Sensemaking and Secure Messaging

Sensemaking is the process of assigning meaning to an unexpected event [42,44]. We observed clear examples of patients trying to make sense of their medical condition through secure message communication. Sensemaking may become problematic in cases where patients believe they are messaging their provider but are actually messaging a triage nurse. The disconnect between who the patient thinks they are messaging and the person who actually reads the message may contribute to mismatches in tone and then to unintended negative impacts on patient engagement. Managing this divide may be difficult, and it will likely depend on the delivery system. Nonetheless, patients need to know ahead of time with whom they are communicating when they engage in secure messaging because the physical and verbal cues present in face-to-face visits, telehealth technologies, and telephone messaging are absent.

The act of a nurse escalating a message to the provider holds clues for us about sensemaking and how to manage it in the context of secure messaging. Better understanding of what triggers the escalation of a message could add to our understanding of how outpatient care teams work together to develop a shared mental model of their patients and the actions needed to help patients be healthy. Similarly, we need better information management tools and policies for helping nurses and physicians respond to messages where a patient is expressing uncertainty or struggling to make sense of their medical situation. Likewise, knowledge is needed of when patients are using secure messaging as a tool to understand their own medical situation versus when patients are trying to connect with their care team so they can collectively make sense of the situation. A potential barrier to improving secure message use for sensemaking purposes is that dealing with messages containing this type of content is unlikely to save system time, just as playing phone tag for days decreases efficiency. We believe it likely that these are the sensemaking-oriented messages that nurses and physicians complain about when they express negative perceptions of communicating with patients by secure message. Given the nature of these messages, a richer communication channel, such as face-to-face or synchronous communication, is better suited. Another model might be to have a secure message in which sensemaking content is detected trigger a nurse message requesting a time to talk with the patient by phone. Regardless, we need better understanding of why patients use secure messaging to communicate complex and ambiguous information and IT communication tools designed to help providers manage this type of information from patients.

### Tone Mismatch and Patient Engagement

Our analysis highlighted concern about mismatch in tone between the messages written by patients and the responses written by their care team. Patient messages in our dataset were generally received and triaged by a nonphysician, and the patients may have been unaware that their physicians were not actually the first people to receive their messages. This potential disconnect may have been a factor in the messages that were tone mismatched. It is also possible that the individuals responsible for triaging patient messages may not have understood the importance of their role in establishing and maintaining rapport with patients over secure message, or that because they are working from a computer (sometimes for long stretches of time) they temporarily forget that they are communicating with a patient who needs their help. We often think of secure messaging as a way to increase patient satisfaction, but if the response patients receive is uninviting or unconcerned, patient satisfaction may decrease. Repeated exposures to tone mismatch could result in patients refusing secure messaging tools, thus creating long-term challenges for organizations wanting to use this tool to communicate with patients.

### Urgent Issues and Patient Safety

Despite a small number of urgent issues raised by patients in our dataset, they did exist. Of our 70 secure message threads, 3 contained an urgent medical matter. This number, while small, demonstrates the need for organizations implementing a secure messaging platform to truly teach patients how to use this tool and be explicit in communicating when and when not to use secure messaging. That said, the VA does inform its patients not to use secure messaging for urgent issues. Yet our findings demonstrate the difficulty inherent in educating patients on how to appropriately use new tools for communicating with their providers. Health care systems using secure messaging may need to revise their business rules to accommodate the need to respond to urgent messages. Many organizations’ business rules are predicated on the assumption that no urgent or emergency messages will be sent via secure message. If even a small percentage of patients continue to use secure messaging for urgent issues, this assumption breaks down and introduces patient safety risks. One solution is to include a first message that must be viewed prior to sending a message that reads something like “If this is a medical emergency, dial 911.” Increasing the number of staff available to handle secure messages quickly as opposed to 24 to 72 hours is another potential solution. Accepting that patients may have a legitimate reason to use secure messaging for urgent matters (for instance, if the clinic phone lines are down and the patient is homebound) is another path forward. In this case, allowing the patient to flag their message as emergent or urgent (with definitions clearly labeled) could be a solution. Regardless, the problem of patients using secure messaging to communicate urgent issues remains and the potential patient safety risks can be serious.

### Unresolved Issues, Patient Safety, and Patient Engagement

The finding that issues raised by patients went unresolved presents a challenge to both patient safety and patient engagement. Our analyses found that approximately 50% of the messages categorized as unresolved (6/11) retained that categorization following our medical record review. The potential implications for patient safety and patient engagement are clear. If a patient does not receive a response to a message, particularly if multiple messages receive no response, patient engagement could suffer. Patient engagement, or activation, is an important quality indicator for health care delivery organizations today. If a message contains an urgent issue and it is unresolved, then patient safety may be at risk. One of the remaining 6 unresolved messages in our data contained an urgent issue, which was surprising. Therefore, both patients and representatives of health care delivery systems need to be vigilant about using secure messaging to ensure patient messages are resolved and patient safety is not at risk.

### Future Considerations

Secure messaging is one channel among many for communication between patients and members of their care team. However, little is known about what is unique about secure messaging as a patient-provider communication channel. Likewise, knowledge of potential harms introduced by features of secure messaging, such as asynchronous interaction and difficulty interpreting emotional cues via electronic communication channels, is limited. Future research studying the strengths and weaknesses of secure messaging should examine not only secure messaging as a new efficiency-enhancing communication channel for patients and their providers, but also the potential negative impacts on patient safety and patient engagement—particularly when patients’ goals and intentions for secure messaging are misaligned with providers’ goals and intentions for this communication platform.

Research questions that emerged from this analysis are as follows. How can health care organizations ensure secure messaging is not contributing to new, unanticipated patient safety concerns? Regarding urgent medical matters, is secure messaging a poor communication channel choice? If so, what are some effective strategies for communicating this to patients to avoid introducing new patient safety issues? If an issue a patient raises via secure message goes unresolved, are new patient safety issues introduced? Similarly, does a tone mismatch between patients and their provider teams result in decreased patient satisfaction or patient engagement? These questions tie back directly to our overarching theoretical frame of complexity science, which considers secure message use as a patient-provider interdependency that is interaction oriented and unpredictably dynamic, and to a forward-looking perspective linking improved secure message use with better patient satisfaction, engagement, and health outcomes.

### Limitations

This study had several limitations that should be considered. Because of the qualitative nature of our study, we analyzed only 70 secure message threads. Given the volume of secure messages sent between patients and their providers today, this is a small number. However, our analysis included all secure messages sent in the time period in which the messages were sent, accounting for all secure messaging communication during that period of time. The purpose of this study was to identify and discuss new considerations for secure messaging as they may relate to key patient outcomes such as safety and engagement, as opposed to providing another detailed description of a large repository of secure messages. We believe the information management and uncertainty management categories and their potential to introduce unanticipated patient safety risks and engagement opportunities are novel contributions that add to the larger conversation of how to effectively use secure messaging platforms in health care delivery.

The research setting could be viewed as a limitation. The secure message triage model used in this VA is not uncommon; however, it may not always reflect the secure messaging implementation and use in other health care systems. While the VA is unique in many ways, the challenges it faces with regard to health IT adoption and use by patients and providers are similar to the challenges other health care delivery systems face.

These data were collected in 2013, and secure messaging communication practices may have evolved since then. We also acknowledge that the volume of messages has risen significantly, which may affect how patients and providers communicate with each other using this tool. Additionally, we acknowledge that the VA has worked since our data were collected on guidelines regarding the appropriateness of using secure messaging to address different issues.

We also acknowledge the limits of drawing inferences about patient safety and risks from viewing secure messages (and medical records) alone. This study did not measure the patient-provider relationship external to the messages. We also did not assess patient preferences about communication with their providers; thus, a tonal mismatch, for example, may not negatively affect the patient-provider relationship if both parties have an existing relationship that is strong and might anticipate or overlook tone mismatch.

Finally, this study focused on examining secure messages, and we acknowledge that potential safety concerns are not as compelling as actual patient safety lapses, or even observed near misses. Future research should take a step further in measuring patient safety risks in secure messaging and develop methods for identifying and verifying concerns raised by patients in secure messages and for acting on them in other processes of care experienced by the patient.

### Conclusions

This analysis provides new insights into the complexity of secure messaging communication between patients and their outpatient care teams. Patients use secure messaging to exchange information, solve problems, build relationships, and make sense of their health or illness with their providers. Understanding the extent to which problems initiated via secure message go unresolved is an important piece of the puzzle for understanding the role of secure messaging in the patient-provider team communication toolbox and the potential for unpredictable negative impacts on secure messaging as a communication channel. Likewise, understanding the frequency with which patients are using secure messaging to communicate urgent medical matters is important, particularly given the potential risk to patient safety. Tone mismatches in care team response to patient secure message content is important to examine further because of their potential to negatively affect the patient-centered goals of health care organizations and the overall experience patients have with their health care providers. The patterns identified in this analysis shed light on potential patient safety concerns, particularly when using secure messaging to address some of the more complex issues patients are raising via secure messaging technologies. Finally, this study generated new questions for secure message use requiring additional examination.

## Abbreviations

IT: information technology
VA: US Department of Veterans Affairs
